# Ethnobotanical study on wild plants used by Lhoba people in Milin County, Tibet

**DOI:** 10.1186/s13002-015-0009-3

**Published:** 2015-03-24

**Authors:** Feifei Li, Jingxian Zhuo, Bo Liu, Devra Jarvis, Chunlin Long

**Affiliations:** College of Life and Environmental Sciences, Minzu University of China, Beijing, 100081 PR China; College of Agronomy and Biotechnology, Yunnan Agricultural University, Kunming, 650201 PR China; Kunming Institute of Botany, Chinese Academy of Sciences, Kunming, 650201 PR China; Bioversity International, Via dei Tre Denari 472/a, 00057 Maccarese, Rome, Italy

**Keywords:** Ethnobotany, Medicinal plants, Lhoba, Tibet

## Abstract

**Background:**

The Lhoba are a small ethnic group, located in the Tibet Autonomous Region of China. Until 1960, their livelihood was predominantly based on swidden agriculture, hunting, and gathering. To investigate and document the plant species used by the Lhoba, ethnobotanical surveys were conducted in three villages of Nanyi Township in Milin County, Tibet, China.

**Methods:**

Ethnobotanical surveys were conducted in three Lhoba villages using key informant interviews and semi-structured interviews. Plants traditionally used by the Lhoba were documented. Data obtained were analyzed through informant consensus factor analysis (F_IC_) to determine the homogeneity of the informants’ knowledge of medicinal plants.

**Results:**

Fifty-nine plant species belonging to 49 genera and 28 families were recorded and collected. Twenty-eight species are ethnomedicinal plants, 29 are local edible plants, and 23 are used for other purposes in Lhoba daily life. The medicinal plant species are used for treating eight categories of illness. Most medicinal plants are herbs (71.4%) or roots (39.2%). Nutrition adjustment (F_IC_ = 0.76) and dermatological infections (F_IC_ = 0.56) showed the highest F_IC_, indicating that the Lhoba had the highest level of agreement about the use of plants for these two illness categories. Fruit is the most frequently used part of the edible plants. Nine edible plant species are used as herbal medicine. Plant species used for other purposes include, six species for fuel, five for dye material, six for religious use, four for timber, two for tobacco substitutes, and one for fodder.

**Conclusions:**

Some traditional technologies and customs of Lhoba, such as dyeing and bamboo weaving, have remained the same for centuries. In contrast, the Lhoba’s knowledge of ethnomedicine has been recently influenced by traditional Tibetan and Chinese medicine, resulting in the loss of traditional knowledge in this sector. In addition, the development of tourism has influenced a change in the Lhoba lifestyle and their production of traditional products. These events signal the need to invest in mechanisms that can enable the Lhoba to benefit from the use of their traditional plant-derived culture and therefore support the continued conservation and use of these important plant resources.

## Background

The southeast area of Tibet is one of the 25 biodiversity hotspots in the world [1]. The area is rich in biological resources due to its subtropical humid and semi-humid climate, which extend over extreme elevational differences. Rich medicinal plant resources are distributed in different geographical areas of the region. The region that Nanyi Village is located in has been regarded as a sacred site, and called “Medicinal Lord’s Valley” by healers [2]. The people living in Milin consist primarily of three ethnic groups: the Tibetan, the Monpa (or Moinba or Menba), and the Lhoba (or Luoba). The Lhoba are distributed in three counties of the Nyingchi (Linzhi) Prefecture: Milin, Medog, and Zayü, and in Lhünzê County of the Shannan Prefecture [3]. Researchers have speculated that the Lhoba might be from the integration of several ancient tribes of the southeastern Qinghai-Tibet Plateau [4,5]. Before the Chinese government recognized and decided on “Lhoba” as their unified name in 1965 [6], each tribe had an independent name and a different dialect, “Bo’gaer”, “Bengni”, and “Miguba” [5,7]. “Lhoba” is derived from pronunciation of which means “southerner” in the Tibetan language”, and has been used to refer to the people living in Lhoyü, Tibet [4]. According to the 2010 census, there are only 3,682 Lhoba in the modern-day Tibet Autonomous Region in China, and Milin County contains the largest population of Lhoba (Bo’gaer tribal group) that lacks a mixed inhabitation with other ethnic groups [8,9]. Before the 1960s, the Lhoba mainly lived on the abundant plant resources in the Tibetan mountain valleys. They practiced swidden agriculture, in addition to hunting and gathering activities. For centuries, these plant resources have provided the Lhoba’s most important source for medicine and food supplements [10,11]. The Lhoba have a rich information base of ethnobotanical knowledge for describing and using these species.

The majority of plant-based chemical compounds, which now provide important components of medicines in the world market, come from traditional medicinal plants through the isolation and analysis of the active components [12]. In many developing countries, up to 80% of the population continue to depend on traditional medicines for their primary health care needs [13]. Many valuable nutritional foods came from traditional foods [14], while the value of wild food plants is very important for cultural and nutritional perspectives [15]. Traditional plant-based knowledge and the plants themselves remain crucial for the development of new drugs, preparation of ethnic food, and other plant based product development [15-18].

The Tibetan region is a hot spot for ethnobotanical studies [19-21], particularly related to Tibetan medicine [22-25]. In recent years, wild edible plants used by Tibetan ethnic groups have become of interest to ethnobotanists. Ju and colleagues identified and recorded the use of over 168 wild edible plant species used by Tibetans in the Shangri-la region in Yunnan Province, China [26]. Kang and colleagues surveyed 81 species of wild food plants used by the Tibetans of Gongba Valley in Zhouqu County, Gansu Province, China [27]. However, to date, the knowledge of medicinal and wild edible plants in the Lhoba communities has been unexplored.

The purpose of this study was to document the traditional ethnobotanical knowledge of the Lhoba, to understand the relationships between the Lhoba and their living environment, and to review the impact of Tibetan culture on this knowledge. We also examined whether the ethnobotanical knowledge of the Lhoba was similar to published information on the Lhoba tribes in neighboring India.

## Methods

### Site description

**Figure 1:**
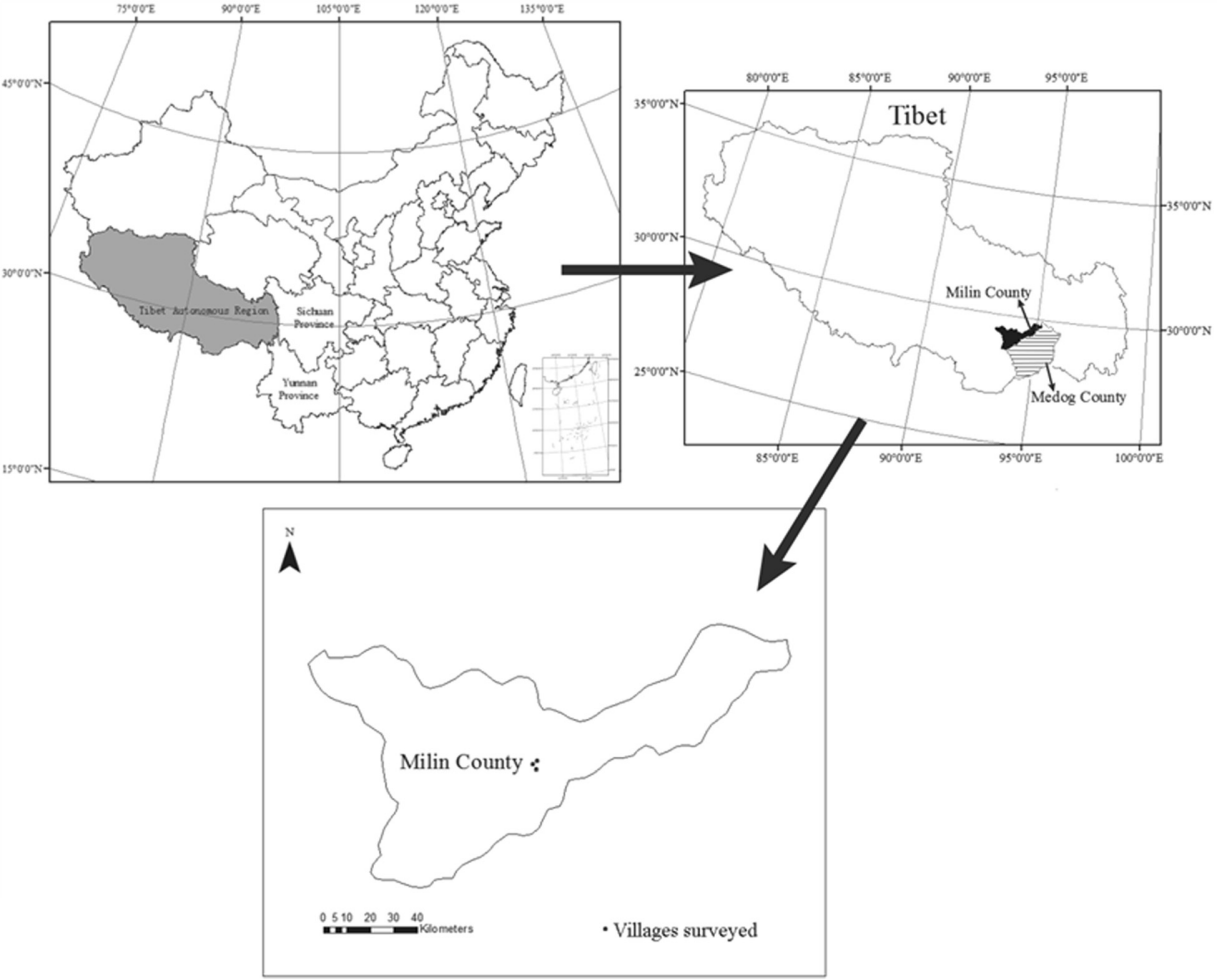
**Location of the study area, Milin County, Tibet, China.**

This study was conducted in September 2012, and July to September 2013, in three villages of Nanyi Township in Milin County: Caizhao Village (N 29°11′,E 94°11′), Qionglin Village (N 29°12′, E 94°12′), and Nanyi Village (N 29°10′, E 94°12′) (Figure 1). Nanyi Township covers a total area of 648.4 km^2^, including 38.7 km^2^ of forest, 140 km^2^ of grassland, and 2.3 km^2^ of glaciers. There are 109 families living in the county with a total population of 515. Milin County lies in the middle river valley of the Yarlung Tsangpo River, with an average altitude of 2,940 m. The average temperature of the coldest month ranges from 0.1°C to 3.2°C, and the average temperature of the hottest month ranges from 12.3°C to 17.4°C. The annual average rainfall is 600 mm, and the average humidity is 66% [28]. Mountain brown soil and dark brown soil are the major types of soil. The vegetation of the area is dominated by a temperate semi-humid monsoon forest.

In the study area, the Lhoba use the Bo’gaer dialect, which belongs to the Tani language branch of the Sino-Tibetan language family of the Tibeto-Burman language [29]. The Lhoba traditional houses are built with bamboo and timber. Three stones lie in the center of the house, with a stone bowl on them, used for cooking food. Staple foods are finger millet (*Eleusine coracana*), rice, corn, and buckwheat. Clothes are usually made from bamboo shells, vines, bearskins, and palm fibers. The traditional belief of the Lhoba is animism; the ghost, god, demon, and elf are not distinguished, and all of them are called “Wuyou” [4]. The Lhoba have two kinds of witches or wizards for divination and to sacrifice to. Recently, Lhoba traditional culture has been deeply affected by Tibetan culture; most young and mid-aged Lhoba speak the Tibetan language or Mandarin Chinese, and Tibetan New Year is their major festival [9].

### Ethnobotanical survey and data collection

The traditional plant-based information was collected through participatory rural appraisal (PRA), direct observation, and semi-structured and key informant interviews [30-33]. Twenty-three respondents with ages ranging from 20 to 65 years were included in the interviews. Informants were asked to give the local names of plants, ailment treated, parts used, cooking or preparation method, and other uses of the plants. Interviews were conducted in the local language by visiting each respondent individually, with assistance from translators and field work guides from the township. Permissions were provided by all participants in this study, including the local Lhoba people. Consent was obtained from the participants prior to this study being carried out. Uses of the plants were grouped into three categories: medicinal, edible, and other uses. Specimens were collected and identified by the authors and deposited in the Herbarium of Minzu University of China (Beijing).

### Data analysis

To estimate the consistency of informants and the extent that the informants agree on the use of certain plant species for the treatment of a given illness or illness category, an informant consensus factor (F_IC_) was calculated for testing homogeneity in informant responses [34]. The formula is:

$$ {\mathrm{F}}_{\mathrm{IC}}=\left({N}_{ur},-,{N}_t\right)/\left({N}_{ur},-,1\right) $$

where N_ur_ is the number of individual plant use-reports for each ailment category, and N_t_ is the total number of species used by all informants for this ailment category. F_IC_ values range from 0 to 1, where higher values indicate higher consensus.

## Results and discussion

**Figure 2:**
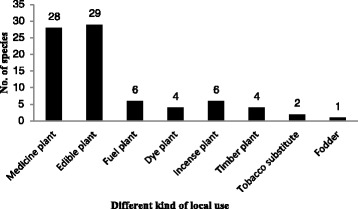
**Plants used for different purposes by the Lhoba ethnic group in Milin County, Tibet, China.**

Ethnobotanical information for 59 plant species belonging to 49 genera and 28 families were collected from the study area (Table 1). These species include angiosperms (54 spp.), gymnosperms (2), pteridophyte (1), algae (1), and lichen (1). Within these plant species, 36 are herbaceous (61%), 14 are shrubs (24%), and nine are trees (15%). According to our survey, 28 species are ethnomedicinal plants, 29 are local edible plants, and 23 are used for other purposes in Lhoba daily life, such as fuelwood (6), dye (5), religious (6), timber (4), tobacco substitutes (2), and fodder (1) (Figure 2).

**Table 1:** **Ethnobotanical inventory of Lhoba in Milin County, Tibet, China**

**Family name**	**Scientific name**	**Local name**	**Habit**	**Part used**	**Local use**
Adoxaceae	*Sambucus adnata* Wall. ex DC.	Ong na nie na san dou ba	Herb	Fruits and roots	Medicine used for treating bruises. Fruits are edible and sweet.
	*Viburnum kansuense* Batal.	Ga ma mi me	Shrub	Fruits	Food.
	*Viburnum nervosum* D. Don	Ji bong	Shrub	Roots	Soaked in alcohol for anti-inflammatory and relieving pain as external medicine.
Apiaceae	*Angelica apaensis* R. H. Shan et C. Q. Yuan	Jia na	Herb	Whole plant	Boiled, used as hypotensive drugs.
Balanophoraceae	*Balanophora involucrata* Hook. f.	Di du guo ya	Herb	Whole plant	Soaked in alcohol or boiled in water for aphrodisiac.
Berberidaceae	*Berberis atrocarpa* Schneid.	Jiu zi ca ma	Shrub	Leaves and fruits	Food (sour taste).
	*Berberis kongboensis* Ahrendt	Jiu zi ca ma	Shrub	Leaves and fruits	Food (sour taste).
	*Berberis pruinosa* Franch.	Sai mang	Shrub	Branches, roots and fruits	Branches and roots are boiled in water and used as medicine for treating diarrhea. Fruits are edible and sour. Fruits and roots are also used for dyeing.
	*Berberis temolaica* Ahrendt	Si sen	Shrub	Roots	Dye plant.
	*Dysosma tsayuensis* Ying	Dong na long dong	Herb	Fruits	Eaten directly as food or boiled in water as medicine for treating gynecological diseases or hematinics.
	*Sinopodophyllum hexandrum* (Royle) Ying	Dong na long dong	Herb	Fruits	Eaten directly as food or boiled in water as medicine for treating gynecological diseases or hematinics.
Bignoniaceae	*Incarvillea lutea* Bur. et Franch.	Di ma bu du	Herb	Roots	Medicine used for hematinics.
Clusiaceae	*Hypericum bellum* Li	Da bu ru ma	Herb	Fruits	Tobacco substitutes.
Compositae	*Ajania tenuifolia* (Jacq.) Tzvel.	Yi lin	Herb	Whole plant	Incense plant
	*Anaphalis nepalensis* (Spreng.) Hand.-Mazz.	A bo	Herb	Whole plant	Tobacco substitute, kindling and fuel
	*Artemisia vestita* Wall. ex Bess.	Can ba	Herb	Whole plant	An important incense plant.
	*Cirsium eriophoroides* (Hook. F.) Petrak	Da ca ma	Herb	Whole plant	Medicine external used for stopping bleeding and reducing the inflammation.
	*Erigeron breviscapus* (Vant.) Hand.-Mazz.	Ra jiang	Herb	Flowers and roots	Boiled or eaten directly, used for treating dyspepsia, headache and kidney deficiency.
	*Leontopodium dedekensii* (Bur. et Franch.) Beauv.	Ba bong bin	Herb	Whole plant	Kindling and fuel.
	*Ligularia rumicifolia* (Drumm.) S. W. Liu	Lang qian niu ba	Herb	Roots	Boiled liquid for treating sore throat as an anti-inflammatory medicine.
	*Senecio scandens* Buch.-Ham. ex D. Don	Gang bu rong ba	Herb	Roots	Boiled for treating cold.
	*Synotis solidaginea* (Hand.-Mazz.) C. Jeffrey et Y. L. Chen	Mi ji dong ba	Herb	Whole plant	Boiled liquid for treating stuffy nose and freckle.
Cupressaceae	*Juniperus squamata* Buch.-Ham. ex D. Don	Ba ma	Tree	Leaves and branches	Fuel, incense and timber plant.
Elaeagnaceae	*Elaeagnus umbellata* Thunb.	Jiu gong/Ran jia	Tree	Fruits	Food (sour and sweet taste), used for treating stomach pain.
	*Hippophae rhamnoides* Linn. subsp. *yunnanensis* Rousi	Da guo	Tree	Fruits	Food (sour and sweet taste), dye plant.
Ericaceae	*Gaultheria wardii* Marq. et Airy-Shaw	Dong gou mi xi	Shrub	Fruits	Food.
	*Rhododendron cephalanthum* Franch.	Da jia bu	Shrub	Leaves and branches	Incense plant.
	*Rhododendron primuliflorum* Bur. et Franch.	Da jia bu	Shrub	Leaves and branches	Incense plant.
Fagaceae	*Quercus aquifolioides* Rehd. et Wils.	Sen nie ya ye	Tree	Fruits and branches	Unshelled and crushed fruits are used for making flat cake. Branches are used for making agriculture tools, weaving tools and fuel.
Grossulariaceae	*Ribes himalense* Royle ex Decaisne	Ong m li	Shrub	Fruits	Food (sour and sweet taste)
	*Ribes laciniatum* J. D. Hooder et Thomson	Ong m li	Shrub	Fruits	Food (sour and sweet taste)
Lamiaceae	*Elsholtzia ciliata* (Thunb.) Hyland.	Bong ga da nang	Herb	Whole plant	Spice plant for making blood sausages.
	*Elsholtzia densa* Benth.	Bong ga da nang	Herb	Whole plant	Spice plant for making blood sausages.
	*Elsholtzia strobilifera* Benth.	Bong ga da nang	Herb	Whole plant	Spice plant for making blood sausages.
	*Phlomis milingensis* C. Y. Wu et H. W. Li	Ou mu ba wa	Herb	Flowers	Nectar
	*Salvia przewalskii* Maxim.	Re nie	Herb	Flowers	Nectar
Lauraceae	*Litsea cubeba* (Lour.) Pers.	De yi	Tree	Fruits	Medicine used for treating stomach disorder and diarrhea. Fruits are fried with pepper as substitute of spices.
	*Litsea pungens* Hemsl.	Ta er	Tree	Fruits	Fried with pepper as spices substitutes.
Melanthiaceae	*Paris polyphylla* Smith	Da bi ri sen	Herb	Roots	Medicine used as a kind of inflammation-relieving hemostatic medicine.
Pinaceae	*Abies forrestii* C. C. Rogers	Song	Tree	Branches	Fuel, used for making barrel and other living appliances
Plantaginaceae	*Veronica anagallis- aquatica* Linn.	Bong ga neng bong	Herb	Whole plant	Food (a kind of vegetable). Medicine used for treating sore throat.
Poaceae	*Fargesia macclureana* Yi	La rang	Herb	Branches	Fuel, thatching, weaving basket and other instruments of labor.
Polygonaceae	*Polygonum hydropiper* Linn.	A er	Herb	Aboveground part	Dye plant
	*Polygonum tortuosum* D. Don.	Ya rong	Herb	Whole plant	Boiled roots used for treating diarrhea. Fodder.
Primulaceae	*Primula sikkimensis* Hook.	Qiu dong ba	Herb	Roots	Boiled for treating diarrhea.
Pteridiaceae	*Pteridium aquilinum* (Linn.) Kuhn var. *latiusculum* (Desv.) Underw. ex Heller	Da wang	Herb	Burgeens	Food (a kind of vegetable)
Ranunculaceae	*Aconitum kongboense* Lauener	Ao mo mu ji	Herb	Roots	Poison for hunting. Grinding roots into power and stick on arrowhead with water to make the arrow poison. Medicine used for paretic analgesia.
	*Batrachium bungei* (Steud.) L. Liou	Xi jiao	Herb	Leaves	Medicine used to wash hair for hair healthy.
	*Coptis teeta* Franch.	Meng ba	Herb	Roots	Medicine used in wound care for stopping bleeding, relieving pain, anti-inflammatory and detoxification properties.
Rhamnaceae	*Berchemia yunnanensis* Franch.	Guo lang	Tree	Fruits	Medicine used for stomach pain
Rosaceae	*Fragaria vesca* Linn.	Yi gei ba qi	Herb	Fruits	Food (sweet taste)
	*Potentilla anserina* Linn.	Ba xi	Herb	whole plant	Incense plant. Roots are cooked with other food material for nutritional supplement.
	*Rosa omeiensis* Rolfe	Ha ji ba bu	Shrub	Fruits	Food (sweet taste) used for treating dysentery and cold.
	*Rubus biflorus* Buch.-Ham. ex Smith	Yi na/Zi ga	Shrub	Fruits	Food (sweet taste), good for kidney.
	*Sorbus thibetica* (Card.) Hand.-Mazz.	Bo lang	Tree	Fruits	Food (sour and sweet taste),eaten after frost for replenishing strength.
Rubiaceae	*Rubia cordifolia* Linn.	Da min	Herb	Whole plant	Dye plant
Rutaceae	*Zanthoxylum bungeanum* Maxim.	Ye ma	Shrub	Fruits	Spices
Usneaceae	*Usnea* spp.	Bi ba beng suo	Herb	Whole plant	Soaked in water, used to wash feet for treating beriberi.
Zygnemataceae	*Spirogyra* spp.	Ni a ji	Herb	Whole plant	Food (making soup)

(Ranked by family names alphabetically, followed by generic and species names).

### Wild medicinal plants

The information for the ethnomedicinal species was recorded, including the botanical names, the local names, the part used, the method of preparation, and the ailments treated. Most medicinal plants are herbs (71.4%). Roots (39.2%) are the most predominantly used part of these medicinal plants, followed by fruits (28.6%), whole plant (28.6%), leaves (3.6%), branches (3.6%), and flowers (3.6%). Results are similar to other ethnobotanical studies of medicinal plants [35], in that the most frequently used part of the plant was the underground part, where higher amount of bioactive compounds than for the other parts are noted [36]. The most commonly used method of preparation was decoction, in which the plant is boiled in water until the water is reduced to more than half its original volume.

Based on the information from the informants, the uses for all reported illnesses for wild medicinal plants are grouped into eight categories [37] (Table 2): dermatological infections/diseases, circulatory system, genito urinary ailments, hair disorders, gastro intestinal ailments, nutrition adjustment, respiratory system disorders, and skeleton muscular system disorders. F_IC_ results for the eight illness categories ranged from 0.3 to 0.76, with the highest for nutrition adjustment (F_IC_ = 0.76; 6 species, 22 use-reports) and dermatological infections (F_IC_ = 0.56; 5 species, 10 use-reports) (Table 2). One of the Lhoba’s important avocations is hunting, which is associated with injuries from accidents. *Dysosma tsayuensis* and *Sinopodophyllum hexandrum* were the most commonly used species for treating gynecological diseases or as hematinics. According to the China Red Data Book, *Dysosma tsayuensis* and *Sinopodophyllum hexandrum* are vulnerable species, *Coptis teeta* is an endangered species, and *Dysosma tsayuensis* is endemic to Tibet [38,39].

**Table 2:** **Ethnobotanical consensus index for traditional medicinal plant use categories**

**Illness category (diseases and disorders)**	**Biomedical terms**	**Number of taxa (N**_**t**_ **)**	**Number of use reports (N**_**ur**_ **)**	**Informants’ consensus index factor (F**_**IC**_ **)**
Dermatological infections/diseases	cuts and wounds	5	10	0.56
Circulatory system	high blood pressure and altitude reaction	2	3	0.5
Genito urinary ailments	sexual weakness, menstrual problems and kidney deficiency	5	8	0.43
Hair problem	hair loss	1	1	--
Gastro-intestinal ailments	diarrhea, stomach pain and dyspepsia	8	11	0.3
Nutrition adjustment	anemia and malnutrition	6	22	0.76
Respiratory system disorders	cold, sore throat and stuffy nose	5	8	0.43
Skeleto muscular system disorders	inflammation and curing traumatic injury	2	3	0.5

Literature studies revealed that the same parts of 12 of the species (43%) collected in this study are also used in Tibetan medicine [40] (Table 3). Three of these species: *Berberis pruinosa*, *Polygonum tortuosum*, and *Potentilla anserina*, are used in Tibetan medicine to treat the same ailments. Seven other species (*Angelica apaensis*, *Dysosma tsayuensis*, *Sinopodophyllum hexandrum*, *Cirsium eriophoroides*, *Erigeron breviscapus*, *Coptis teeta*, *Usnea* spp.) had partial uses similar with Tibetan medicine. And the two remaining species (*Rosa omeiensis* and *Sambucus adnata*) are used for different uses by the Lhoba than in Tibetan medicine. Although some studies indicated more Lhoba living in adjacent Indian, only two speices, *Litsea cubeba* [11] and *Coptis teeta* [18,19,22] were used in the same ways by the Lhoba and these tribal peoples [11,41-44].

**Table 3:** **Comparison of Lhoba plant use and Tibetan use of reported medicinal plants**

**Family name**	**Species name**	**Habit**	**Part used**	**Lhoba use**	**Tibetan use** **[** 40 **]**
Adoxaceae	*Sambucus adnata* Wall. ex DC.	Herb	Roots	Bruises.	Eczema, edema^3^
	*Viburnum nervosum* D. Don	Shrub	Roots	Injury, pain	
Apiaceae	*Angelica apaensis* R. H. Shan et C. Q. Yuan	Herb	Whole plant	Hypertension	Skin diseases, nosotoxicosis^2^
Balanophoraceae	*Balanophora involucrata* Hook. F.	Herb	Whole plant	Aphrodisiac.	
Berberidaceae	*Berberis pruinosa* Franch.	Shrub	Branches and roots	Diarrhea.	Diarrhea, grasserie, and flu^1^
	*Dysosma tsayuensis* Ying	Herb	Fruits	Gynecological diseases and anemia.	Gynecological diseases, nephropathy^2^
	*Sinopodophyllum hexandrum* (Royle) Ying	Herb	Fruits	Gynecological diseases and anemia.	Gynecological diseases, nephropathy^2^
Bignoniaceae	*Incarvillea lutea* Bur. et Franch.	Herb	Roots	Anemia	
Compositae	*Cirsium eriophoroides* (Hook. F.) Petrak	Herb	Whole plant	Bleeding and inflammation.	Edema, bleeding, epistaxis, menorrhagia^2^
	*Erigeron breviscapus* (Vant.) Hand.-Mazz.	Herb	Flowers and roots	Dyspepsia, headache, and kidney deficiency.	Ophthalmalgia, headache^2^
	*Ligularia rumicifolia* (Drumm.) S. W. Liu	Herb	Roots	Sore throat and inflammatory	
	*Senecio scandens* Buch.-Ham. ex D. Don	Herb	Roots	Cold	
	*Synotis solidaginea* (Hand.-Mazz.) C. Jeffrey et Y. L. Chen	Herb	Whole plant	Stuffy nose and freckle	
Elaeagnaceae	*Elaeagnus umbellata* Thunb.	Tree	Fruits	Stomach pain	
Lauraceae	*Litsea cubeba* (Lour.) Pers.	Tree	Fruits	Stomach disorder and diarrhea.	
Melanthiaceae	*Paris polyphylla* Smith	Herb	Roots	Wound and inflammation	
Plantaginaceae	*Veronica anagallis-aquatica* Linn.	Herb	Whole plant	Sore throat	
Polygonaceae	*Polygonum tortuosum* D. Don.	Herb	Whole plant	Diarrhea	Diarrhoea, gastricism^1^
Primulaceae	*Primula sikkimensis* Hook.	Herb	Roots	Diarrhea	
Ranunculaceae	*Aconitum kongboense* Lauener	Herb	Roots	Poison	
	*Batrachium bungei* (Steud.) L. Liou	Herb	Leaves	Hair loss	
	*Coptis teeta* Franch.	Herb	Roots	Bleeding, pain, inflammatory and detoxification properties	Intestinal diseases, anthrax, dysentery, pyogenic infection^2^
Rhamnaceae	*Berchemia yunnanensis* Franch.	Tree	Fruits	Stomach pain	
Rosaceae	*Potentilla anserina* Linn.	Herb	Whole plant	Hyposthenia	Hyposthenia ^1^
	*Rosa omeiensis* Rolfe	Shrub	Fruits	Dysentery and cold	Skin diseases, arthralgia^3^
	*Rubus biflorus* Buch.-Ham. ex Smith	Shrub	Fruits	Kidney deficiency	
	*Sorbus thibetica* (Card.) Hand.-Mazz.	Tree	Fruits	Hyposthenia	
Usneaceae	*Usnea* spp.	Herb	Whole plant	Beriberi and ulcer	Tracheitis, mastitis, ulcer, pneumonia, hepatitis, toxic fever^2^

(Ranked by family names alphabetically, followed by genus and species names).

1: Local use coherent with Tibetan use; 2: Local use coherent with Tibetan use partially; 3: Local use not coherent with known Tibetan use.

### Wild edible plants

Twenty-nine wild plant species are commonly used as food in Lhoba society, including 12 herbs, 10 shrubs, and 7 trees. The most frequently used part is the fruit (19 species, 65.5%). This is similar to the percentage use of the fruit of wild edible plants in the Sikkim Himalaya [45]. The Lhoba depended on wild fruit such as *Rosa omeiensis*, *Rubus biflorus*, *Sorbus thibetica*, or *Ribes himalense* for vitamines and nutrients nutrition. Reliance on fruit from wild edible species may be related to the low productivity of cultivated fruit trees of the Lhoba. The Lhoba reported that eating too much *Sambucus adnata* (Ong na nie na san dou ba) causes headaches. Most fruit are eaten directly, except *Quercus aquifolioides* (Sen nie ya ye), *Litsea cubeba* (De yi), *Litsea pungens* (Ta er), and *Zanthoxylum bungeanum* (Ye ma). Fruits of *Quercus aquifolioides* “Sen nie ya ye” are the Lhoba’s traditional food. The Lhoba remove the nutshell and astringency, crush the nuts, and bake the flower from the nuts as cakes. “De yi”, “Ta er,” and “Ye ma” are used as important spices or spice substitutes, and are boiled or stir-fried with other vegetables or meat. The Lhoba also mix spices with salts, yogurt, crushed vegetables, or mushrooms, and then dip steamed bread in this mixture. Bamboo shoots are usually collected and eaten from wild bamboo species, such as *Fargesia macclureana*, and are usually made as sour bamboo shoots for longer storage and a change in taste. Out of 29 wild edible species, nine are also used as herbal medicine. *Veronica anagallis-aquatica*, for example, is usually boiled as a vegetable and could be used for treating sore throats.

### Plants used for other purposes

Dye plants are significant in the Lhoba’s livelihoods. The Lhoba have rich experience in extracting dye from plants and in dye technology. The exchange of dye plants has an important position in trade between the Lhoba and the Tibetans, because dye plants are the main raw material for Tibetan Buddhists to dye their clothes. The investigation revealed that *Berberis pruinosa* (Sai mang), *Hippophae rhamnoides* subsp. *yunnanensis* (Da guo), *Berberis temolaica* (Si sen), *Rubia cordifolia* (Da min), and *Polygonum hydropiper* (A er) are common dyeing species. Boiling the fruits or roots of “Sai mang”, “Si sen”, or “Da guo” with thread for one hour produces a thread that can be dyed yellow. “Da min” can turn yellow thread to red. “A er” can be used for dyeing wools to black. The Lhoba people put mashed “A er” into a gourd cask with wool, then mix with hot water, and hang the sealed gourd cask over fireplace; after fermenting for 4–5 days, the dyed black wool is taken out and dried in the sunlight. *Polygonum hydropiper* and *Rubia cordifolia* are also used as dye plants called “Chhum-gon” in Monpa and “Tamen” in Adi, respectively [46].

**Figure 3:**
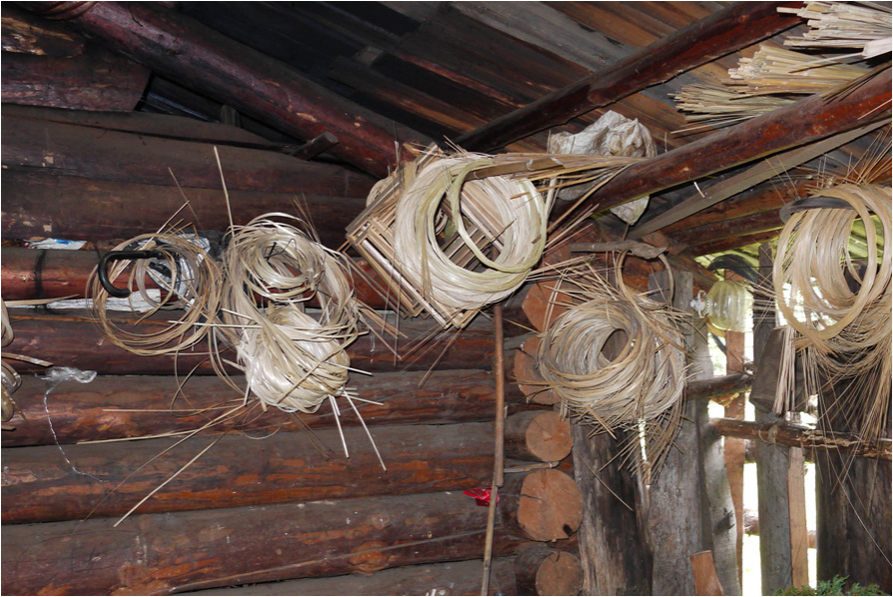
**Inside a Lhoba house and Lhoba bamboo weaving.**

*Abies forrestii, Juniperus squamata*, and *Quercus aquifolioides* are the main timber species that the Lhoba used for building their houses and are also used to make agricultural tools or daily-life utensils. For example, *Abies forrestii* can be used to make barrels and *Quercus aquifolioides* for “Da luo”, is used to make wooden shovels for digging. Bamboo weaving of the Lhoba in Nanyi is very famous. The common bamboo species is *Fargesia macclureana*. Bamboo canes are cut into thin ribbons and used for weaving baskets, mats, cages, bowls, rain gear, bows, arrows, and some other common livelihood items (Figure 3). The bamboo weaving handicraft skills of the Lhoba are similar to the Yak Pastoralists [34].

The Lhoba culture has been deeply impacted by Tibetan culture. The Lhoba have animistic beliefs, but they have adopted many religious rituals of Tibetan Buddhism, such as burning offerings [9]. *Potentilla anserina*, *Artemisia vestita*, *Ajania tenuifolia*, *Juniperus squamata*, *Rhododendron cephalanthum*, and *Rhododendron primulaeflorum* are used as incense sources, which play an important role in the religious activities of the Lhoba. Plants used for fuelwood plants include three tree species: *Quercus aquifolioides Juniperus squamata*, and *Abies forrestii*, and three herbaceous species: *Anaphalis nepalensis*, *Leontopodium dedekensii*, and *Fargesia macclureana*. The three herbaceous species are used as good kindling. Most Lhoba, both male and female, have the habit of smoking, and use *Anaphalis nepalensi* and the fruits of *Hypericum bellum* as substitutes for tobacco.

The study revealed that the traditional uses of plant species of the Lhoba in Milin County are closely related to their living environment. For example, palms are mentioned in earlier studies of Lhoba culture in Medog County, a county adjacent to Minlin County, but at lower elevations with a tropical environment [47]. Studies for Medog County mentioned that, in food shortage situations, the Lhoba in Medog County extracted starch from palm’s stems as an important wild food source, and used tropical fruits such as banana, citrus, and betel nuts [48,49]. Our investigation found no record of the Lhoba using palms or tropical fruits, which may be due to the differences in climate and thus available possible species to be exploited as food [50].

The Lhoba transfer their plant-based knowledge from one generation to the next through elders by oral tradition, without any written documents. The influence of tourism, socioeconomic development, the small group size, and a lack of interest shown by the young generation have seriously threatened this non-literate ethnic culture [11,51]. Recently, better accesses to markets have provided the younger generation with sufficient food and medicine, removing the need for wild plant harvest. In addition, our results show that increased publicity for and availability of Tibetan and Chinese medicines has affected the indigenous knowledge of the Lhoba, especially the youth who put more value on the medicines that pharmaceutical companies or medicine buyers are purchasing from the community, such as *Ophiocordyceps sinensis*. Recent tourism has also affected the passing on of Lhoba traditional culture. Lhoba run businesses often serve as guides in the adjacent tourism area. The influenced of tourism culture, used for attracting tourists has resulted in tourist guides providing incorrect or unreliable information on Lhoba culture. For example, Lhoba guides told tourists that *Hippophae rhamnoides* subsp. *yunnanensis* was the holy tree in traditional Lhoba culture, while other Lhoba, not in the tourist business, stated that this claim was incorrect.

## Conclusions

This study documented traditional ethnobotanical knowledge of the Lhoba in Nanyi Township, Milin County, Tibet. Fifty-nine wild plant species were found to be used in traditional medicines, food, dyeing technologies, and religion. These species mainly came from the surrounding areas. Some of these materials are important trade items in local Tibetan and Lhoba markets. The Lhoba in Nanyi use the same plant species for dyes and had similar bamboo weaving handcraft as tribes in adjacent areas in India. In contrast the Lhoba’s use of ethnomedicinal species has been deeply influenced by traditional Tibetan medicine and Chinese medicine. This study reported less plant species compared to other ethnic communities in Tibet. This may be due to the small size of the Lhoba population. The improved access to imported goods from outside their community and the development of tourism has changed the Lhoba lifestyle and production structure. These events signal the need to invest in mechanisms that can enable the Lhoba to benefit from the use of their traditional plant-derived culture and therefore support the continued conservation and use of these important plant resources.
